# The use of games in the classroom to promote mental health knowledge and healthy attitudes in adolescents: a systematic review

**DOI:** 10.3389/fpsyt.2025.1622099

**Published:** 2025-09-01

**Authors:** Juan P. Sanabria-Mazo, Adrián Pérez-Aranda, Carilene Armas-Landaeta, Estíbaliz Royuela-Colomer, Corel Mateo-Canedo, Itxaso Cabrera-Gil, Alicia Monreal-Bartolomé, Javier García-Campayo, Yolanda López-del-Hoyo

**Affiliations:** ^1^ Teaching, Research, and Innovation Unit, Parc Sanitari Sant Joan de Déu, Sant Boi de Llobregat, Spain; ^2^ Center for Biomedical Research in Epidemiology and Public Health (CIBERESP), Madrid, Spain; ^3^ Department of Clinical and Health Psychology, Autonomous University of Barcelona, Cerdanyola del Vallès, Spain; ^4^ Faculty of Medicine, University of Zaragoza, Zaragoza, Spain; ^5^ Department of Basic, Developmental, and Educational Psychology, Autonomous University of Barcelona, Cerdanyola del Vallès, Spain; ^6^ Department of Psychology and Sociology, University of Zaragoza, Zaragoza, Spain; ^7^ Institute of Health Research of Aragon (IIS Aragón), Miguel Servet University Hospital, Zaragoza, Spain; ^8^ Research Network on Chronicity, Primary Care and Health Promotion (RICAPPS), Zaragoza, Spain

**Keywords:** adolescents, classroom, game-based interventions, public mental health, systematic review

## Abstract

**Background:**

Many adolescents face significant mental health challenges, including anxiety, depression, and substance-related and addictive disorders, with a growing need for school-based preventive strategies. Traditional classroom interventions often struggle to engage adolescents, prompting the exploration of game-based approaches to improve mental health knowledge and foster healthier attitudes.

**Objective:**

This systematic review evaluates the effectiveness of game-based interventions implemented in classroom settings to enhance mental health knowledge and promote positive attitudes among adolescents.

**Methods:**

A comprehensive search was conducted across Medline, PsycINFO, Web of Science, and SCOPUS, yielding 1,152 articles. After screening, 17 studies were included, most using cluster randomized controlled trial designs. These interventions addressed topics of mental health such as substance-related and addictive disorders, anxiety, and depression, employing playful activities like video games, virtual reality simulations, quizzes, and collaborative tasks.

**Results:**

The interventions showed positive effects on knowledge acquisition and attitude change, with high satisfaction reported by participants. Those programs targeting alcohol and tobacco use were particularly effective in improving knowledge and attitudes. However, the quality of evidence varied, and long-term effects were not widely assessed due to a lack of follow-up in most studies. Some interventions had a limited impact on self-efficacy and behavior change.

**Conclusions:**

Game-based classroom interventions show promise in promoting mental health literacy and healthier attitudes among adolescents. While short-term outcomes are encouraging, more rigorous studies with extended follow-up periods are necessary to understand their long-term effectiveness. Future interventions should focus on practical skill development and personalization to maximize impact.

**Systematic review registration:**

https://www.crd.york.ac.uk/PROSPERO/, identifier CRD42024531914.

## 1 Introduction

Adolescence, a critical phase in human development, is marked by significant biological and social changes that have lasting effects on long-term well-being (1). Mental health in adolescents is an area of growing concern: a recent report by the United Nations (2) indicated that 1 in 7 children and teens are impacted by mental health conditions. The neurodevelopmental transformations during this period, combined with the unique challenges of adolescence—such as evolving relationships with parents, exploration of social and sexual roles, identity formation, and planning for the future—heighten the risk of psychological distress. In some cases, this distress can manifest in mental health conditions such as anxiety, depression, and substance-related and addictive disorders (3). Recent evidence suggests that the COVID-19 pandemic has exacerbated mental health issues within this demographic (4–6), with substance-related and addictive disorders emerging as particularly concerning due to their association with long-term negative consequences, including a higher risk of mood disorders, academic difficulties, and social impairments (7).

This life stage constitutes a period of vulnerability to mental health issues, which represents a propitious time for prevention and intervention (8). Universal preventive strategies delivered in schools, designed to reach all adolescents irrespective of their individual risk factors or symptomatology, present a hopeful avenue for fostering mental well-being (9). By targeting a diverse range of adolescents, regardless of their initial susceptibility, these universal interventions hold the potential to mitigate the onset of problematic behaviors while concurrently enhancing overall mental health outcomes by increasing knowledge and modifying attitudes. Such school-based strategies can effectively reach a broader spectrum of adolescents, potentially reducing barriers associated with seeking professional help and minimizing stigma surrounding mental health treatment (10).

Traditionally, interventions delivered in the classroom involved educational programs designed to provide knowledge about mental health disorders, reduce stigma, and prevent risky behaviors related to addictive substances. However, while some classroom-delivered interventions have shown a certain degree of effectiveness (11), these programs are not exempt from challenges. A primary hurdle lies in the capacity of traditional educational models to truly engage and positively impact the target audience. Adolescents often perceive conventional teaching methods as outdated or disconnected from their daily experiences, which can diminish the impact of these interventions (12, 13).

To overcome the limitations of traditional approaches, it is crucial to adopt innovative strategies that bridge the gap between educational content and the lived experiences of adolescents. One promising method involves incorporating interactive elements, such as games, that directly address adolescents’ concerns and interests. Some game-based interventions are characterized by the incorporation of game elements but cannot fully qualify as stand-alone games (14). In contrast, other interventions, including serious games, educational games, and game-based learning strategies, are explicitly designed to educate players, enhance their skills, or enrich their knowledge (15).

By integrating dynamics such as points, levels, and rewards, gamification transforms educational content into a format that feels less like traditional learning and more like an engaging activity (15, 16). This game-based approach not only boosts participation and sustained interest but also allows for personalized learning experiences tailored to individual preferences and learning styles. Although previous systematic reviews have identified positive effects of gamified interventions in specific populations, such as adolescents with obesity (17), adolescents with attention-deficit hyperactivity disorder (18), or individuals from the general population (19), the specific impact of game-based interventions on promoting mental health knowledge and fostering healthy attitudes in adolescents has not been systematically explored.

Given the gaps in current research, an updated systematic review is needed to examine the available evidence on this topic. To date, the existing literature has not determined the extent to which game-based interventions can improve adolescents’ knowledge of mental health problems or encourage positive attitudes toward mental health. The objective of this study was to conduct a comprehensive systematic review examining various forms of game-based interventions implemented within classroom settings. These interventions are hypothesized to be effective and engaging tools for promoting mental health awareness. The reviewed interventions were required to include playful activities and be designed to enhance mental health knowledge dissemination and behavior change across diverse mental health conditions in adolescent populations. By scrutinizing the gamified approaches utilized and their corresponding outcomes, this study aims to provide valuable insights into the potential of playful interventions as effective tools for fostering mental health awareness and encouraging positive behavioral changes in adolescents.

## 2 Method

### 2.1 Study design

This systematic review was conducted following the Preferred Reporting Items for Systematic Reviews and Meta-analysis (PRISMA) guidelines (20). The review protocol was registered in the Prospective Register of Systematic Review (PROSPERO) with the identification code CRD42024531914.

### 2.2 Data sources and search strategy

A search strategy using Medline, PsycINFO, Web of Science, and SCOPUS was conducted. This search strategy combined terms related to population (adolescent OR teenager OR youth OR juvenile OR young OR minor), games (game OR gaming OR gamified OR gamification), interventions (intervention OR program OR treatment OR therapy OR trial), and classrooms (classroom OR school OR education institution). All searches were performed based on titles, abstracts, and keywords. The bibliographic database searches are detailed in **Supplementary Table 1**.

The search terms were selected considering the search strategies in previous systematic reviews on game-based interventions (21–23). The following limits and filters were activated in all databases, if possible: (1) publication date (from inception until 2024), (2) document type (only articles), and (3) languages (English and Spanish). In addition, gray literature—such as dissertations, conference abstracts, or reports—was excluded to ensure methodological consistency across included studies. The reference list of included studies was examined through a reverse citation search.

### 2.3 Eligibility criteria

The eligibility criteria were selected using the “Population”, “Intervention”, “Comparison”, “Outcomes”, and “Study” (PICOS) approach (24):

- [P] Population: Adolescents aged between 11 and 18 years. Studies that did not specify age or did not indicate the age range of the sample were excluded. No additional exclusion criteria were established for the population.
- [I] Intervention: Those delivered in the classroom that incorporate playful activities to promote mental health knowledge and behavior change directed at diverse mental health conditions. Interventions could be fully or partially gamified, cooperative or competitive, and conducted face-to-face or using a digital device (if performed in the classroom). Blended interventions (i.e., a combination of face-to-face and digital device sessions) were also included. Interventions that did not include playful elements or were not conducted in the classroom were excluded.
- [C] Comparison: To explore all available evidence in the literature, single-, two-, or multi-arm interventions were included. No inclusion or exclusion criteria were established for controls.
- [O] Outcomes: Mental health-related outcomes were explored in this systematic review, such as mental health problems, knowledge about mental health problems, attitudes towards mental health problems, frequency of risk behaviors, or symptomatology, among others. Participants’ satisfaction with the interventions was also explored. Studies with outcomes not directly related to mental health were excluded.
- [S] Study design: Randomized controlled trials (RCTs), non-RCTs, and open trials of any length of follow-up were included. Pilot studies were also included if they followed one of the previous study designs. No trials were excluded based on publication status.

### 2.4 Study selection

Duplicate articles in the databases were automatically removed by Mendeley. Then, 2 authors (CAL and APA) independently screened all articles in Rayyan QCRI. Abstracts and titles were screened to identify those relevant to the research question. When insufficient information was available to determine eligibility, full articles were reviewed. The selected articles were examined for their reference lists to identify additional relevant studies that might not have appeared in the initial database search. Initial disagreements among the authors were resolved by consensus. No additional reviewer was needed to resolve the initial disagreement.

### 2.5 Quality appraisal

The quality appraisal (QA) of the studies was evaluated independently by 3 authors (CAL, APA, and JPSM) using the National Heart, Lung, and Blood Institute (NHLBI) tools for quality assessment (25). Considering the characteristics of the studies included in this systematic review, two tools were used: one for controlled intervention studies, and another for single-arm studies.

The total score range was 0–14 for the “Quality Assessment of Controlled Intervention Studies” tool: good QA (11-14), fair QA (6-10), and low QA (≤ 5); and 0–12 for the “Quality Assessment Tool for Before-After Studies with No Control Group” tool: good QA (9-12), fair QA (5-8), and low QA (≤ 4). The answer options were scored with 1 when the criterion was met and with a 0 when the criterion was not met, could not be determined, was not applicable, or was not reported. Discordances in quality rating were resolved through discussion between the authors.

### 2.6 Data extraction and synthesis

A data extraction form was developed based on the Centre for Reviews and Dissemination templates. Data collected included information on authors, publication date, country of study, study design, sample size, setting, outcomes, and results. In addition, the name, reference(s), objective, format, and games of the interventions included in this systematic review were extracted. A narrative synthesis was carried out to describe the main characteristics of game-based interventions, the quality of evidence, and the relationship of the findings within and between the included studies. A summary of each study and its main features was provided in the tables.

## 3 Results

### 3.1 Selection and inclusion of studies

**Figure 1:**
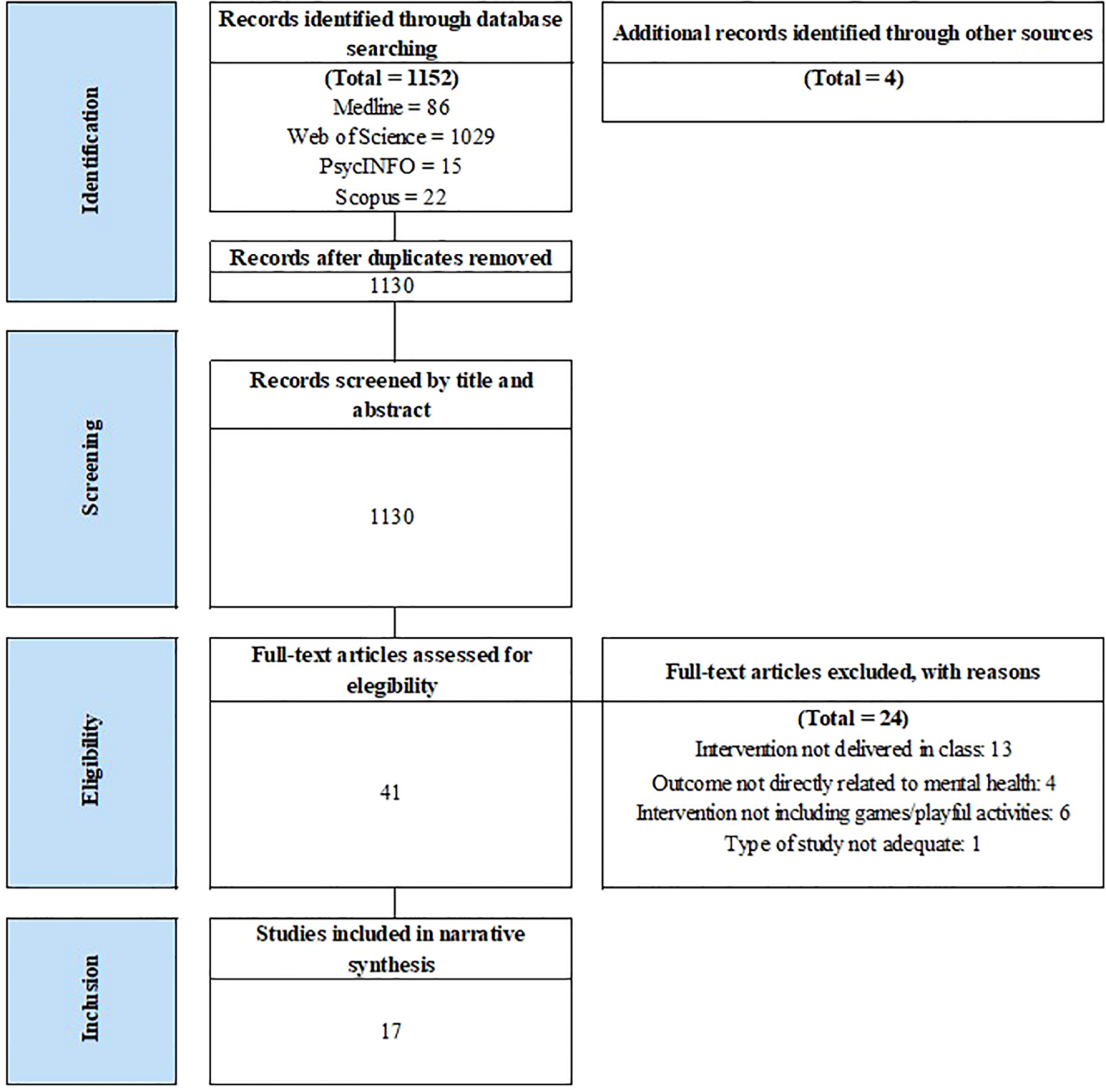
PRISMA flowchart from record identification to study inclusion. Source: PRISMA, https://www.prisma-statement.org/prisma-2020-flow-diagram, reproduced under CC BY 4.0 license.

As shown in **Figure 1**, the initial database search yielded a total of 1,152 published articles. In addition, 4 articles were included from other resources (i.e., reverse citation search). After removing duplicates, 1,130 titles and abstracts were reviewed, of which 41 were chosen for full-text review. Finally, 17 articles were included in this systematic review.

### 3.2 Summary of the findings

Among the 17 studies, the cluster randomized controlled trial design was the most common (n = 12), where the whole classrooms were randomly assigned either to the intervention or control condition. Only six studies included a follow-up assessment. The sample sizes ranged from 40 to more than 14,000 adolescents, and participants’ ages ranged from 11 to 18. Gender distribution was balanced in most cases, although one study was conducted on a female-only education center. Regarding the outcomes, most studies (n = 12) included some measure of knowledge acquisition (regarding the aspects that the intervention targeted), attitudes, and intentions, among others. In addition, eight studies reported users’ opinions and/or satisfaction with the intervention. These outcomes were predominantly measured using *ad hoc* questionnaires—some previously employed in other studies, others specifically developed for the current research—while only 7 studies used some validated instruments. The description of each study can be found in **Table 1**.

**Table 1:** Description of the 17 studies reviewed.

Reference	Study design	Sample	Intervention	Outcome(s)
Bezençon et al. (26)	Cluster randomized controlled trial (mixed methods): · Escape addict (*n* = 75) · Control (*n* = 80) Baseline and post-intervention assessments.	*N* = 202 adolescents from 4 schools in Switzerland · 49% females · Mean age not reported (range: 12-15)	Escape Addict	· Factual knowledge^1^ · Risk perception^2^ · Behaviors (time spent talking about addictions, time spent playing videogames, and privacy settings on social networks)^1^ · Educational achievement^1^ · Opinions about the program^1^
Dietrich et al. (27)	Single-arm study with 3 subsamples: · “skeptics” (*n* = 128) · “risky males” (*n* = 121) · “good females” (*n* = 122) Baseline and post-intervention assessments.	*N* = 371 adolescents from Catholic schools in Queensland (Australia) · 51% females · 14.77 years old (range: 14-16)	GO: KA	· Attitude toward binge drinking^2^ · Behavioral intention^2^ · Alcohol knowledge^2^ · Opinions about the program^1^
Dietrich et al. (28)	Single-arm study (mixed methods): · Blurred Minds (*n* = 373) Baseline and post-intervention assessments.	*N* = 374 adolescents from 9 intervention schools in Queensland (Australia) · 58% females · 15.2 years old (range: 14-16)	Blurred Minds	· Attitude toward binge drinking^2^ · Social norms^2^ · Self-efficacy^3^ · Behavioral intention^2^ · Alcohol knowledge^2^ · Opinions about the program^1^
Durl et al. (29)	Cluster randomized controlled trial: · Blurred Minds: brief program pilot (*n* = 115) · Blurred Minds: comprehensive program (*n* = 336) · Control schools (*n* = 368) Baseline and post-intervention assessments.	*N* = 819 adolescents from 6 intervention schools in 2 Australian states · 47% females · Age not reported (range not reported)	Blurred Minds	· Attitude toward binge^2^ drinking · Social norms^2^ · Self-efficacy^2^
Galli et al. (30)	Quasi-experimental longitudinal study: · Intervention group (*n* = 167) · Control group (*n* = 112) Baseline and post-intervention assessments.	*N* = 279 adolescents studying in 6 Italian sports high schools · 39.9% females · 17.6 years old (range not reported)	I am on top!	· Doping intention^2^ · Self-regulatory efficacy to resist social pressure^1^ · Moral disengagement^2^ · Doping knowledge^2^
Guldager et al. (31)	Cluster randomized controlled trial: · Experimental group (*n* = 183) · Control group (*n* =195) Baseline, post-intervention, and 6-week follow-up assessments.	*N* = 378 adolescents from 11 Danish public schools. · 50.8% females · 15.67 years old (range 15-18)	VR FestLab	· Drinking refusal self-efficacy^3^ · Drug refusal skills^3^ · Knowledge/awareness^1^ · Communication skills^3^ · Social support^1^ · Susceptibility to peer pressure^3^ · Outcome expectations^3^
McMahon and Hanrahan (32)	Cluster randomized controlled trial: · LifeMatters (*n* = 20) · Normal timetable (*n* = 20) Baseline and post-intervention assessments.	*N* = 40 disengaged students from a YMCA vocational school in Kingston (Australia) · 12.5% females · 16.7 years old (range: 16-17)	LifeMatters	· Relatedness^3^ · Social anxiety^3^
Perry et al. (33)	Cluster randomized controlled trial: · Project MYTRI (*n* = 6,365) · Wait-list control (*n* = 7,698) Baseline and post-intervention assessments.	*N* = 14,063 students in the 6th and 8th grades from 32 schools in Delhi and Chennai (India). · 43% males · Mean age not reported (range: 11-14)	Project MYTRI	· Psychosocial risk factors^2^ · Tobacco use and intentions^2^
Rundle-Thiele et al. (34)	Single-arm pilot study with 2 subsamples: · all-boys school (*n* = 131) · all-girls school (*n* = 102) Baseline and post-intervention assessments.	*N* = 233 adolescents from Catholic schools in Queensland (Australia) · 44% females · Mean age not reported (range: 14-18)	GO: KA	· Attitudes^2^ · Behavioral intentions^2^
Rundle-Thiele et al. (35)	Cluster randomized controlled trial: · GO: KA (*n* = 942) · Control schools (*n* = 578) Baseline and post-intervention assessments.	*N* = 1520 adolescents from Catholic schools in Queensland (Australia) · 45% females · Mean age not reported (range: 14-18)	GO: KA	· Affective attitude^2^ · Instrumental attitude2 · Subjective norms^2^ · Intention toward binge drinking^2^ · Knowledge^2^
Sidhu et al. (36)	Quasi-experimental longitudinal study: · Project EX (*n* = 321) · Standard care control (*n* = 303) Baseline and post-intervention assessments.	*N* = 624 adolescents studying in 4 different schools in Delhi (India) · 42% females · 16.8 years old (range: 14-19)	Project EX	· Tobacco use behavior^1^ · Tobacco prevention and cessation^1^ · Opinions about the key activities in the program (meditation, yoga, healthy breathing, and quitting tobacco, among others)^1^
Stein-Seroussi et al. (37)	Cluster randomized controlled trial: · ACTION (*n* = 123) · Comparison condition: videos + scripted discussion questions (*n* = 138) Baseline, post-intervention, and 3-month follow-up assessments.	*N* = 264 adolescents from 14 schools in Kentucky, North Carolina, and Ohio (United States) · 38% of females · 16.1 years old (range: 14-18)	ACTION	· Tobacco use behavior^1^ · Nicotine dependence^3^ · Stages of change^2^ · Motivation to quit smoking^1^ · Exposure index^1^ · Dosage^1^ · Participants’ perceptions of the program^1^ · Program fidelity^1^
Stigler et al. (38)	Cluster randomized controlled trial: · Project MYTRI (*n* = 4,009) · Wait-list control (*n* = 4,360) Baseline and post-intervention assessments.	*N* = 8,369 6^th^ and 8^th^ grade students from 32 schools in Delhi and Chennai (India) · 48.4% females · Mean age not reported (range: 11-14)	Project MYTRI	· Psychosocial risk factors^1^ · Tobacco use and intentions^1^
Sussman et al. (39)	Cluster randomized controlled trial: · Project EX (*n* = 139) · Project EX + school-as-community (SAC) component (*n* = 120) · Standard care control (*n* = 76) Baseline, post-intervention, and 3-month follow-up assessments.	*N* = 335 smokers studying in 18 continuation high schools in California (United States) · 36% females · 16.8 years old (range: 14-19)	Project EX	· Tobacco use behavior and intentions^2^ · Nicotine dependence^3^ · Stages of tobacco use^2^ development and cessation · Opinions about the overall program and each session (helpfulness, interest, likability, and informativeness, among others)^1^
Tuijnman et al. (40)	Cluster randomized controlled trial: · Moving stories (*n* = 99) · No intervention (*n* = 86) Baseline, post-program, 3-, and 6-month follow-up assessments.	*N* = 185 adolescents studying in 4 high schools in the Netherlands · 45.4% females · 13.43 years old (range: 12-15)	Moving stories	· Mental health literacy^2^ · Depression stigma^3^ · Opinions about the program^1^
Wodarski (41)	Cluster randomized controlled trial: · TGT (*n* = 526) · Traditional instruction, 1-week course (*n* = 361) · No instruction (*n* = 384) Baseline, post-intervention, and 1-year follow-up assessments.	*N* = 1,271 adolescents studying in 5 schools in the metropolitan area of Georgia (United States) · Gender not reported · Age not reported (range not reported)	TGT	· Knowledge of alcohol^1^ · Attitudes about alcohol use^1^ · Current alcohol use^1^ · Impulsive behavior^1^
Zarshenas et al. (42)	Cluster randomized controlled trial: · Microlearning (*n* = 125) · Gamification (*n* = 129) · Control group (*n* = 124) Baseline and post-intervention assessments.	*N* = 378 students in the 7^th^, 8^th^, and 9^th^ grades of 3 high schools in Fars (Iran). · 100% females · Mean age not reported (range: 13-15)	Gamified educational program	· Anxiety state^3^ · Anxiety trait^3^

^1^The variable was measured using an *ad hoc* questionnaire designed for the study; ^2^the variable was measured using an *ad hoc* questionnaire that had already been used in previous studies; ^3^the variable was measured using validated questionnaires or parts of validated questionnaires (e.g., Drinking Expectancy Questionnaire – Revised, Drinking Refusal Self-Efficacy Questionnaire – Revised, Brief Assessment Tool of the Life Skills Training, Alcohol Misuse Prevention Knowledge Questionnaire, He Social Interaction Anxiety Scale, Basic Measure of Psychological Needs, 6-Item Fagerström Tolerance Questionnaire, Dutch Depression Stigma Scale, Social Distance Scale, Children’s Depression Inventory, and the State-Trait Anxiety Inventory).

The studies reviewed tested the effects of 12 interventions that included some kind of game in the classroom to increase knowledge and/or promote healthy attitudes related to mental health: four interventions aimed at promoting responsible drinking; three focused on preventing/reducing tobacco use among adolescents; two programs targeted different types of substance-related and addictive disorders (doping, tobacco, alcohol, cannabis, and screen use); two focused on reducing anxiety; and one was addressed at increasing knowledge about depression and reducing its associated stigma. While some interventions were delivered in a single session (n = 3), most of them (n = 8) included at least four that took place once per week during consecutive weeks. The playful activities delivered in the classroom were diverse: videogames in which the students had to make decisions regarding the behavior of the main character and see the consequences (n = 2), virtual reality games that simulated social situations and bodily sensations (n = 2), interactive games and competitive quizzes to increase knowledge (n = 6), and a collaborative digital escape room to learn about different types of risky behaviors (n = 1). A description of the interventions, detailing the playful activity that each one included, can be found in **Table 2**.

**Table 2:** Interventions included in the systematic review.

Name of the intervention	Reference(s)	Aims	Format	Game(s)
ACTION	Stein-Seroussi et al. (37)	Teaching smoking cessation and abstinence skills.	6 sessions (50 minutes each). Period of administration not reported. Each session consists of games, during which participants accumulate points across the length of the program.	Students participated in different interactive games. Between sessions, they practiced skills (e.g., contacting each other for support or using relaxation techniques) that emphasized the progressive nature of quitting.
Blurred Minds	Dietrich et al. (28) Durl et al. (29)	To influence adolescents’ knowledge and inform their perceptions surrounding alcohol.	5-lesson intervention delivered in 1 school day. The program features a suite of gamified resources to engage and deliver messages surrounding alcohol. Each lesson focuses on different learning outcomes.	The students played different games (VR house party, alcohol trivia show, the perfect pour, Deso driver, dumb driver, bar master, and BACToZero) and practical activities (beer goggles, passing out, keeping it classy, disco dance, legal Q&A, power of marketing, myths-norms).
Escape Addict	Bezençon et al. (26)	To raise awareness, provoke reflections, and eventually lead to behavior change related to risky and addictive behaviors (tobacco, alcohol, cannabis, and screen use) and their consequences.	1.5h session, including 15 min of instruction at the beginning and debriefing at the end. The class is split up into self-selected groups of 4–6 pupils. Each group receives a digital tablet.	The game starts with a narrative audio recording setting the stage of the experience: the class is locked down until pupils conduct a set of 4 investigations. Each investigation has its scenario based around a teenager who has some issues related to risky or addictive behaviors and requires teams to perform certain tasks to correctly give answers to quizzes. The experience provides different immersive interactions through the tablets (e.g., augmented reality and 360° camera). In addition to the tablets, the experience makes use of several physical artifacts to provide a richer experience. Finally, once all the teams have solved the 4 investigations, the class is brought together to solve the final puzzle. The session ends with a debrief led by the facilitator.
Game On: Know Alcohol (GO: KA)	Dietrich et al. (27); Rundle-Thiele et al. (34); Rundle-Thiele et al. (35)	To influence attitudes and behavioral intentions towards moderate drinking.	6-module intervention delivered to a year-level cohort in an auditorium. The intervention consisted of 6 modules: (1) Game on! Risky ride; (2) Know the effects of alcohol; (3) Game on! Drink aware; (4) Know standard drinks; (5) Game on! Stay in control; and (6) Strategies for drinking moderately in social settings.	The students played different games, including classroom games about risky activities, drinking awareness, and the importance of staying in control. 3 modules consisted of games and the other 3 consisted of group activities and discussions. A total of 9 activities, 4 online activities (3 games and 1 quiz), and 5 experiential activities.
I am on top!	Galli et al. (30)	To promote self-regulation processes in the prevention of the use of doping.	4 sessions (90 minutes each), spread over about a month during school lessons and relying on a trained sports psychologist. In the third session, the scores obtained in the game are discussed. This would likely have been an interactive session wherein participants could share their experiences, ask questions, and express their thoughts and concerns related to their scores.	The game presents the everyday life of a track and field athlete who experiences different situations leading up to running in an important competition. The main character interacts with his girlfriend, parents, coach, and teammate with whom he faces different decisions and challenges related to the possibility of using doping substances. The protagonist has the option to make different decisions and, based on them, the climax of the story presents different scenarios (e.g., the athlete decides to not use doping and wins the race; the athlete denounces the teammate; the athlete consumes banned substances, etc.).
LifeMatters	McMahon & Hanrahan (32)	Teaching trust, communication, and problem-solving abilities to enhance relatedness and decrease social anxiety.	10 sessions (2 hours each) delivered over 2 weeks. Sessions contained worksheets, discussions, and activities related to mental skills. There were different types of games: (1) “icebreakers”, (2) “desinhibiters”, (3) “trust and empathy”, and (4) “initiative”.	Students played games in a predefined sequence. First, they participated in “icebreaker” games to develop rapport with other students; second, in “desinhibiters” games to learn that it is okay to receive help from others; third, in “trust and empathy” games to learn that it is okay to depend on others; and fourth, “initiative” games to improve effective communication, cooperation, and problem-solving.
Moving stories	Tuijnman et al. (40)	It targets recognition, knowledge of help-seeking options and treatments, and first aid skills, specifically for depression in youth.	1-week program delivered in the classroom with 3 parts: (1) an introduction lesson, (2) a single-player, mobile 3D video game, and (3) a contact session with someone who has experienced a depressive disorder.	In the game, they interact with Lisa, who shows signs of depression. The adolescents are asked to help her by performing 5 actions each day; some of these actions have a positive effect on their relationship with Lisa, and others have a negative effect. During the day, they received messages from Lisa with feedback on their actions. After 5 days of playing, they were able to see a final scene in which Lisa explained that she got help and thanked the players for their help. In the classroom, adolescents could play the game individually but in the same period as their classmates to allow for joint playing time and shared feedback moments in the game.
Project EX	Sidhu et al. (36) Sussman et al. (39)	To prevent and reduce tobacco use among adolescents.	8 sessions delivered over 6 weeks. Each session includes a “talk show” and/or a game.	Students create a menu of possible categories and order questions regarding the dangers of passive smoking as a group competition.
Project MYTRI (Mobilizing Youth for Tobacco-Related Initiatives in India)	Stigler et al. (38); Perry et al. (33)	To prevent and reduce tobacco use among adolescents.	The first-year curriculum included 7, 70-minute classroom sessions. The classroom curricula involved games, competitions, and other activities that were designed to be fun and interactive. Activities were conducted in small groups of 10 to 15 students and were led by student-elected peer leaders who received training before classroom sessions. Teachers also received prior training and participated in implementing activities.	Game boards and game cards. As an extension of the classroom activities, competitions were also held within and between schools (i.e., intraschool and interschool activities) and included model building (crafting a 3-dimensional model of a tobacco-free school) and street play (an extended, culturally appropriate role play to practice refusal skills) competition for the 6th and 8th graders.
Team, Games and Tournament (TGT)	Wodarski (41)	Providing information that emphasizes behavioral objectives that lead to responsible drinking practices.	Four-week educational program including a 50-minute daily session. TGT was divided into 3 components: (1) learning about alcohol through discussion and participatory activities, (2) preparing for the tournament, and (3) competing in teams in a quiz game.	The tournament games consist of short-answer questions to assess and reinforce the knowledge gained in class. Each team member competes individually against other 3 students of comparable achievement levels. The points gained by each member determine if they will remain in the same category or change to a higher or lower one. The points gained by each member are added and presented the next day.
Unnamed gamified educational program	Zarshenas et al. (42)	Reducing anxiety.	Students interact using a website in which the contents of the intervention (positive mental imagery, diaphragmatic breathing, replacing negative thoughts with positive ones, and training of assertiveness) are presented.	The website was based on gamification and made use of game elements, including scoring, standings, and badges of honor. After each session, a short test was considered for the students where they competed with their peers based on their obtained scores.
VR FestLab	Guldager et al. (31)	Improving the refusal self-efficacy of adolescents who face social pressures to drink alcohol.	A single session that took place during an in-class teaching session. The session started with a gameplay introduction and exploration phase of about 45 min. The class was divided into groups of a maximum of 13 students who played the game at any one time. Thereafter, a structured reflection of the experiences was moderated by a trained study assistant.	The user is confronted with several behavioral options (i.e., different forms of responding to peer pressure), where peers encourage the user to choose either to drink alcohol or soft drinks/water, dance, play, or interact with others. The user’s decision to drink alcohol, the type of drinks (low or high in alcohol concentration), and the time between consuming alcoholic drinks is computed by underlying software to provide visual feedback on blood alcohol concentration to enhance the user’s knowledge and awareness regarding the effects of different alcoholic drinks on the physical state of the body.

### 3.3 Study quality

The overall quality of the studies included was rated as fair. No studies were rated as good, while 4 presented low quality. The risk of bias was mainly due to a lack of blinding in controlled studies, while single-arm studies did not report information on the sample’s representativeness and sample size calculation. The quality assessment of the included studies is detailed in **Supplementary Table 2**.

### 3.4 Effects of the interventions on mental health promotion

#### 3.4.1 Drinking behaviours

Four interventions were specifically designed to target risky drinking behaviors: Blurred Minds (28, 29), Game on: Know Alcohol (GO: KA; 27, 34, 35), Team-Games-Tournaments (TGT; 41), and VR FestLab (31). Another one, Escape Addict, included content related to alcoholism along with other types of addictive disorders (26). The effects of these interventions are summarized in **Table 3**.

**Table 3:** Effects of the intervention.

Reference	Post-intervention effects	Follow-up effects	User satisfaction
Bezençon et al. (26)	The intervention boosted correct answers (*p* <.01). Other variables (e.g., risk perception, gaming time) showed no significant effects. Higher-achieving students benefited more in knowledge acquisition. Gender and self-reported enjoyment had a limited impact on the effect of the intervention.	–	Pupils enjoyed the program (5.68/7). They found the situations presented realistic, engaged actively in team discussions and understood the game questions well. Qualitative interviews confirmed their enjoyment and most felt they learned something valuable.
Dietrich et al. (27)	Compared to “skeptics”, “risky males” and “good females” showed the significant greatest change in drinking attitudes (*p* <.001) and intention towards binge drinking (*p* <.01) at post-treatment. No significant differences were identified between the 2 groups in alcohol knowledge.	–	The subgroup “good females” reported the highest satisfaction with all program components (*p* <.001) and “skeptics” lowest program satisfaction with all program components (*p* <.001).
Dietrich et al. (28)	Participants showed significant greatest change in drinking attitudes (*p* <.001), self-efficacy (*p* = .023), and knowledge (*p* <.001) at post-treatment. No significant change in social norms and intention towards binge drinking was identified at post-treatment.	–	The qualitative results indicated high levels of satisfaction among the participants with the implementation of this program through virtual reality.
Durl et al. (29)	Participants in the comprehensive and brief program scored significantly higher on attitudes (*p* <.001 and *p* <.05, respectively), social norms (*p* <.05 in both cases), and self-efficacy (*p* <.001 in both cases) at posttreatment.	–	Participants stated they were “somewhat satisfied” or “very satisfied” with each of the online and offline activities and games included in the interventions.
Galli et al. (30)	Students’ intention to dope decreased over time for the “intervention group” and increased over time for the “control group” (*p* = .05; *np^2^* = 0.014). The same happened to knowledge about doping (*p* = .002; *np^2^* = 0.036). Self-efficacy and moral disengagement significantly decreased over time in both groups.	–	–
Guldager et al. (31)	The intervention had no significant effects compared to the control group.	The intervention had no significant effects compared to the control group.	–
McMahon & Hanrahan (32)	The intervention group experienced a significant reduction in social anxiety (*p* = .03, *np^2^* = 0.16) and a significant increase in relatedness (*p* = .05, *np^2^* = 0.14) at post-treatment. No significant differences were found between groups when controlling for social anxiety or relatedness.	–	–
Perry et al. (33)	–	Over time, cigarette and bidi smoking rates increased by 68% in the control group but decreased by 17% in the intervention group. Intentions to smoke rose by 5% in the control group and fell by 11% in the intervention group. Intentions to chew tobacco decreased by 12% in the control group and by 28% in the intervention group. Gender and grade-level interactions were significant for all variables.	–
Rundle-Thiele et al. (34)	Boys and girls perceived drinking to excess more negatively after participating in the intervention (*p* <.05 and *p* <.001, respectively). In contrast to boys, girls also reported better behavioral intentions after the intervention (*p* <.05).	–	–
Rundle-Thiele et al. (35)	Participants in the intervention group reported significantly higher scores on affective attitudes (*p* <.05), instrumental attitudes (*p* <.01), and knowledge (*p* <.001) at post-treatment. No significant differences were identified between the 2 groups in subjective norms and intention towards binge drinking.	–	–
Sidhu et al. (36)	Compared to the control group, participants in the intervention group revealed a significant prevention effect (*p* = .05) at post-treatment. Within the model, condition effect (*p* = .03), living condition (*p* = .02), and age (*p* = .04) were statistically significant. No significant cessation effect was observed between the 2 groups.	–	Opinion on the program was favorable. The average satisfaction with the program was 7.77 (*SD* = 2.28), and the average likeability of the program activities ranged from 7.43 (*SD* = 2.38) to 8.65 (*SD* = 1.76). In total, 27% of participants reported that “Project EX” helped to strengthen their commitment to stay tobacco free.
Stein-Seroussi et al. (37)	No significant differences were observed between the 2 groups in achieving abstinence at post-treatment using per protocol analytical approach and intention to treat. No significant effects of the program on nicotine dependence, stages of change, motivation to quit smoking, and exposure index were found.	Compared to the control groups, participants in the intervention group were more likely to achieve abstinence at follow-up (*OR* = 4.44), but not at post-treatment. This difference was statistically significant using the per protocol analytical approach, but not the intention to treat.	Compared to the control group, participants assigned to the ACTION program had significantly higher acceptability scores, for all scales: situation management (*p* = .01), cessation skills (*p* <.001), program likeability (*p* = .05), and teacher likeability (*p* = .01), although scores were generally high for both groups.
Stigler et al. (38)	While tobacco use remained unchanged, fewer students in the intervention group intended to smoke in the next year (*p* = .02). Additionally, this group demonstrated enhanced knowledge of tobacco’s health effects (*p* <.01), perceived greater negative social consequences (*p* = .04), showed reduced social susceptibility to smoking (*p* = .03), viewed tobacco use as less acceptable, particularly among peers (*p* <.01), and had better awareness of tobacco control policies (*p* <.01), among other findings.	–	–
Sussman et al. (39)	28% said that the program helped them quit tobacco use completely, 42% said it helped them reduce their tobacco use with the intention of quitting completely, 10% said it helped them reduce, but without the intention of quitting, 14% said they had not reduced, but the class helped them decide to quit in the near future, and 6% said it helped them to maintain a quit attempt they had started at the beginning of class.	A 30-day abstinence rate of 30% vs. 16% for controls, with an odds ratio of 2.21 (*p* <.05). Other analyses of the adjusted rates indicated that the addition of the SAC component did not improve the cessation rates over the clinic alone and that the results did not vary as a function of gender, ethnicity, age, or type of tobacco use combination.	Opinion on the program did not differ by condition and was very favorable 8.18 (*SD* = 1.56). The game session was well-rated (*mean* = 7.41, *SD* = 2.52). Meditation sessions presented the highest ratings.
Tuijnman et al. (40)	Compared with the control group, participants in the intervention group experienced reductions in personal stigma (*p* = .04). No significant differences between the study groups were observed in any other outcome.	Compared with the control group, participants in the intervention group experienced reductions in personal stigma in the 3-month follow-up (*p* = .04). Contrary to expectations, the experimental group presented a reduction in first aid confidence in the 6-month follow-up compared with the control group (*p* = .01). No significant differences between the study groups were observed in any other outcome.	92% of the adolescents indicated they would recommend the game, while only 66% indicated they would recommend the contact session. 77% understood the game, and 68% thought they had learned something from it.
Wodarski (41)	The TGT group experienced significant improvements in knowledge of alcohol use (*p* <.05). Drinking behavior decreased in the experimental group (*p* <.05). The consequences of drinking were also reduced in the TGT group (*p* <.05), while they experienced a significant change in their attitudes towards alcohol compared to the other groups (*p* <.05) and reduced their impulsivity (*p* <.05).	All the effects observed in the post-test assessment were maintained in the 1-year follow-up.	–
Zarshenas et al. (42)	Gamification was more effective than the control group in the reduction of anxiety. However, no significant difference was found in the effectiveness of the 2 intervention methods.	–	–

Most studies reported positive findings regarding knowledge acquisition: five (26–28, 35, 41) observed significant increases in knowledge about alcohol (e.g., low-risk alcohol consumption levels or consequences of drink driving), while one found no effects on this outcome (31). Attitudes related to drinking were assessed in six studies, all of which found significant positive changes (27–29, 34, 35, 41).

Drinking intentions were assessed in four studies, but only one reported a significant positive change in their subsample of females (34). Self-efficacy was evaluated in three studies: the two studies testing Blurred Minds (28, 29) reported positive findings, while Guldager et al. (31) found no significant effect of VR FestLab on self-efficacy. Other outcomes that experienced significant improvements post-intervention were social norms in Blurred Minds (29), and drinking behavior and impulsivity in TGT (41). The remaining outcomes did not experience significant changes (e.g., risk perception, subjective norms, communication skills, social support, and susceptibility to peer pressure).

Only two studies included follow-up assessments regarding the long-term effects of the interventions: Wodarski (41) found that the changes experienced after the TGT intervention were maintained after one year, while no significant effects were found after six weeks, in the case of VR FestLab (31).

#### 3.4.2 Tobacco use

Three interventions were designed to prevent tobacco use: ACTION (37), Project EX (36, 39), and Project MYTRY (33, 38). Again, the Escape Addict program also included content related to smoking prevention (26). The summary of these findings can be found in **Table 3**.

The effects on knowledge acquisition were assessed in Escape Addict and Project MYTRY, both with positive outcomes (26, 38). Project MYTRY’s effect on attitudes (e.g., perceiving tobacco use as acceptable) was also evaluated with significant changes after the 1-year assessment (38), as well as its effect on intentions to smoke or chew tobacco, both in the 1-year and 2-year assessments (33, 38).

Three interventions targeted quitting smoking as a main outcome: while ACTION produced no significant changes (37), Project EX produced higher rates of cessation than a control condition (39), as well as preventive effects (36). Project MYTRY did not produce an effect in the 1-year assessment, but in the second year, cigarette and bidi smoking rates increased by 68% in the control group but decreased by 17% in the intervention group (33). Other outcomes, such as motivation to quit smoking, exposure to tobacco, or stages of change, were assessed in one study, but none of them experienced a significant change in the intervention group (37).

Long-term effects were assessed for three of these interventions: after three months, the ACTION program achieved a significant rate of abstinence compared to the control group (37); similarly, Project MYTRY increased its effect in the 2-year assessment, finding significant cessation rates that were not present in the previous evaluation (33). Project EX, on its part, maintained its effects in the 3-month follow-up assessment.

#### 3.4.3 Other substance-related and addictive behaviours

Two studies addressed risky behaviors associated with different types of addictive disorders: Galli et al. (30) observed that the I am on top! program had a significant impact on knowledge regarding doping and decreased doping intentions compared to the control group. No effects on self-efficacy or moral disengagement were found. On the other hand, Bezençon et al. (26) also reported that the Escape addict program increased the proportion of correct answers by around ten percentage points on average and had a positive impact on the variety of interlocutors with whom the themes of the intervention were discussed, which could be related to a change in attitudes. Risk perception and other behaviors did not experience significant changes. As shown in **Table 3**, no follow-up assessments were included in these 2 studies.

#### 3.4.4 Anxiety

Two interventions addressed anxiety: LifeMatters produced a significant increase in relatedness, as well as a reduction in social anxiety, although both effects were not significant when including the other variable as a covariate in the model (32). In the same line, the intervention tested by Zarshenas et al. (42) reduced anxiety, but not more than the active control condition. These studies did not include any measures of knowledge, attitudes, or intentions. As shown in **Table 3**, the long-term effects of the interventions were not assessed.

#### 3.4.5 Depression

Only 1 study focused on teaching about depression to reduce its associated stigma (40): the Moving Stories program did not produce significant changes in knowledge regarding depression (e.g., symptom recognition), and help-seeking intentions, as well as first aid intentions, were also not improved. However, it did produce a significant decrease in stigma that was maintained in the 3-month follow-up assessment, but not after six months. Other outcomes did not experience significant changes after the intervention: first-aid confidence (which experienced a significant decrease in the 6-month follow-up evaluation), help-seeking behavior, and first-aid behavior. The summary of these findings can be found in **Table 3**.

### 3.5 User satisfaction with the intervention

Eight studies reported users’ opinions about the program, and these were favorable in all cases (see **Table 3**): Escape Addict obtained a 5.68 out of 7, and students provided positive feedback about the contents and dynamics of the intervention. GO: KA, on its part, presented moderate levels of satisfaction (average of 3 out of 5), with one subsample (“good females”) showing more favorable opinions towards it. The Blurred Minds program received positive feedback from the participants in two studies (28, 29). Project EX had an average of around 8 points out of 10 in two studies (36, 39), and the game session was well-rated (7.41/10), although the meditation session was the most liked one. ACTION received better acceptability scores than the control intervention, although they were high for both groups (37). Finally, as reported by Tuijnman et al. (40), 92% of the adolescents who participated in the study indicated they would recommend Moving Stories.

## 4 Discussion

The identification of effective interventions with innovative approaches is necessary to awaken the interest, motivation, and commitment of adolescents toward their physical and mental health (43). This systematic review examined, for the first time, the efficacy of game-based classroom interventions to promote knowledge and healthy attitudes related to mental health among adolescents. Although effects varied by intervention type and context, findings from the 17 included studies (mostly from cluster-randomized controlled trial designs) suggest that these interventions are potentially effective resources for improving mental health knowledge and modifying risk behaviors. They also provide evidence about adolescents’ satisfaction with the content, dynamics, and methodology of these programs.

Despite notable heterogeneity in content, format, and targeted outcomes, certain features appear to enhance intervention effectiveness. Interventions incorporating interactive and immersive elements—such as role-playing scenarios or decision-making with real-time feedback—tended to show stronger post-intervention effects (e.g., Blurred Minds, Escape Addict, Moving Stories, I Am on Top!). Another key factor for sustained impact is multiple sessions with repeated exposure, which seems crucial for maintaining long-term effects (e.g., Project MYTRI, TGT). Similarly, combining game-based activities with guided discussions (e.g., GO: KA, Project MYTRI, LifeMatters) appears more effective than interventions relying solely on self-guided play. Finally, peer interaction also plays an important role in enhancing learning experiences: team-based components boost motivation and engagement, while competitive elements can further reinforce knowledge and behavior change.

For what concerns to those interventions aimed at reducing alcohol consumption, the four of them had positive effects in terms of knowledge acquisition and attitude change. Consistently, programs such as Blurred Minds (28, 29) and GO: KA (27, 34, 35) achieved significant improvements in alcohol knowledge and attitudes towards responsible drinking. However, changes in drinking intentions and self-efficacy were less consistent. Among these interventions, no significant changes were found in outcomes such as risk perception, subjective norms, communication skills, social support, and susceptibility to peer pressure. The lack of change in some outcomes may suggest that these programs need additional components that reinforce the practical skills and confidence of adolescents to apply what they have learned in real-life situations. In addition, most of the studies did not include long-term assessments, which limits the ability to determine the persistence of the effects detected over time.

Two of the three interventions aimed at preventing tobacco use, i.e., Project EX (36, 39) and Project MYTRI (33, 38), also showed improvements in knowledge and changes in attitudes toward tobacco use. Nevertheless, the effects on smoking cessation were heterogeneous, with some studies reporting higher abstinence rates (36, 39) and others finding no significant differences (37). Follow-up results from Project MYTRI (33, 38) showed sustained effects on reducing smoking intention. The variability detected in these interventions could be due to differences in the implementation, duration, and cultural context of the programs, as well as the level of support provided.

On the other hand, the two interventions focused on other substance-related and addictive disorders and the three interventions focused on mental health problems such as anxiety and depression indicated heterogeneous findings. For instance, the I am on top! (30) program succeeded in reducing doping intention and increasing knowledge about its risks but did not have a significant impact on self-efficacy. Similarly, the Moving Stories (40) program reduced depression’s stigma after treatment and at three months, although it did not significantly improve other aspects, such as knowledge about depression or intentions to seek help. Before including covariates in the model, the LifeMatters (32) program produced a significant increase in relatedness and a reduction in social anxiety. Escape Addict (26), which consists of a escape room implemented in the classroom, was effective for improving literacy towards substance-related and addictive disorders, but had no impact on other outcomes such as risk perception of gaming time. More evidence is needed to determine the efficacy of these interventions.

To date, game-based interventions have shown a diverse approach to addressing different health problems in adolescents (17, 18) and in the general population (19). As observed in the studies included in this systematic review, game-based interventions delivered in the classroom have varied according to format and activities, including videogames, virtual reality, interactive games, and collaborative competitions. Over the past three decades, classrooms have evolved significantly, moving from traditional paper-and-pencil activities and whiteboard illustrations to environments equipped with internet connectivity, social media, computer applications, and various technological adaptations. These advancements have likely influenced the design and delivery of game-based interventions. With the vast array of technology available today, it is crucial to continue exploring how to develop games that can successfully engage adolescents while also educating them on important topics such as mental health (44). As the digital landscape grows, researchers should focus on optimizing game design to ensure these interventions are not only engaging but also effective in promoting behavioral change.

### 4.1 Future research

This systematic review highlights the need for further research on the effects of game-based interventions implemented within classroom settings to promote knowledge and healthy attitudes related to mental health in adolescents. So far, most of the studies published in the literature have focused on exploring the benefits of these programs to prevent or intervene in different substance-related disorders (e.g., drinking behaviors, tobacco use, and doping). However, it would be pertinent for future lines of research to explore the efficacy of these interventions in addressing other prevalent mental health problems in adolescents, such as affective or eating disorders, among others (11). Additionally, it is crucial for such studies to explore the long-term effects of interventions targeting adolescents, particularly regarding their impact on knowledge, attitudes, and healthy behaviors.

Identifying potential mediating or moderating factors that may influence the effectiveness of game-based interventions could be key in customizing programs to meet the unique needs of different adolescent groups. Personalization of interventions seems essential to improve the effectiveness of school-based programs (45). Considering factors such as the age, gender, and socioeconomic context of adolescents, among others, could help to design interventions more in line with their needs and interests (46–48). In this regard, exploring diversity emerges as a fundamental aspect of understanding how game-based interventions can be better adapted to meet adolescents’ needs. For instance, Bezençon et al. (26) observed that gender and academic performance played a role in knowledge acquisition. Future research should investigate how diversity-related factors, such as socioeconomic status, race, ethnicity, gender identity, and cultural background, might influence the effectiveness of these programs. Recognizing these elements is essential to developing inclusive and culturally sensitive interventions that can be applied to diverse adolescent populations. Therefore, it is crucial to ensure that these interventions are accessible and relevant to all adolescents, regardless of their background or identity.

For educators, it is considered essential to create interventions that not only inform but also actively involve adolescents through interactive elements and relevant contexts. It would also be necessary for public policies to support the integration of these programs into the school curriculum, providing the necessary resources for their implementation and continuous evaluation. In addition, training educators in the use of these tools could contribute to maximizing their impact (49, 50). Encouraging the consistent practice of acquired skills over time, as well as adapting game content to be resonant with adolescents’ daily experiences could be elements that contribute to increasing their impact and sustainability (15, 16). This, along with the integration of playful elements that promote collaboration and mutual support, could facilitate the creation of more inclusive and motivating learning environments (51). Finally, assessing how different game elements, such as narrative, interactivity, and rewards, influence mental health outcomes could provide valuable information for designing effective interventions. All these improvements could help maximize the potential of games as intervention tools in promoting mental health among adolescents.

### 4.2 Limitations

One limitation of our review is the lack of a meta-analysis. While this approach was considered, it was deemed not feasible for several reasons. Primarily, there is a lack of standardized and validated operational definitions for mental health outcomes in game-based interventions. This heterogeneity in both outcome conceptualization and measurement tools significantly reduced comparability across studies, limiting the potential for data synthesis. Furthermore, outcomes such as knowledge, intentions, and attitudes were measured inconsistently across studies. Specifically, only five studies reported the necessary data, and the wide variety of instruments used rendered the data non-comparable. Additionally, the small number of available datasets would result in low statistical power and unreliable pooled estimates.

Another limitation is the generally low or fair quality of the studies included in this systematic review. The characteristics of the programs (i.e., number of sessions, intervention components, and measures) and targets (i.e., addictive behaviors, depression, and anxiety) were heterogeneous. For what concerns to outcomes, most of them were assessed using *ad hoc* questionnaires which, although sometimes showing acceptable internal consistency, lacked validation—thereby reducing the precision and reliability of the findings. Furthermore, in several cases, the description of the games used was incomplete, limiting the ability to analyze the contribution of specific program components. Only one-third of the studies included follow-up assessments, which hinders understanding of the long-term effects of these interventions. Taken together, these limitations suggest that it is still premature to draw strong conclusions about the effects of classroom-based game interventions to promote mental health among adolescents.

The variability in cohort characteristics across the studies included (e.g., periods of implementation, regional differences, and adolescent population variations) may have influenced the results. Such factors, including social, cultural, and educational changes over time, could limit the generalizability of the findings. Also, studies from the earliest available research were included in the systematic review to offer a comprehensive overview of the evidence on this topic. While this approach enabled the capture of the field’s evolution, it may also present a limitation, as older studies may vary significantly in methodology, context, and outcomes, potentially impacting the overall interpretation of the findings. Moreover, this systematic review included only studies published in English or Spanish, so other relevant evidence could have been excluded.

One last limitation of this review is the limited identification of theoretical frameworks underpinning the interventions. Most studies did not explicitly report the psychological theories guiding their design, which hinders the understanding of the causal mechanisms through which game elements might influence mental health outcomes. This gap limits the ability to draw conclusions about why certain intervention components may be more effective than others. Future research should place greater emphasis on grounding interventions in established theoretical models, which would help clarify the mechanisms of change and inform the development of more effective and targeted game-based strategies.

### 4.3 Conclusion

Game-based interventions represent a promising strategy to address adolescent mental health challenges, particularly in relation to substance-related and addictive disorders. This systematic review found evidence supporting their effectiveness in improving knowledge and attitudes toward alcohol and tobacco use, as well as in reducing the intention to consume these substances. Although further research is needed in areas such as anxiety and depression, the high levels of satisfaction and engagement reported by adolescents highlight the acceptability of these interventions in educational settings. Based on these findings, we recommend that teachers consider integrating evidence-informed game-based activities into health education curricula; that intervention designers align game content with specific psychological targets and employ validated outcome measures; and that policymakers support the implementation and evaluation of these programs by providing resources and training in schools. These recommendations can guide the development of more rigorous, theory-driven interventions and promote mental health literacy among adolescents.

## Data Availability

The original contributions presented in the study are included in the article/**Supplementary Material**. Further inquiries can be directed to the corresponding author.
