# Supplementary material for: The use of games in the classroom to promote mental health knowledge and healthy attitudes in adolescents: a systematic review
Source: Front Psychiatry. 2025 Sep 1;16:1622099. doi: 10.3389/fpsyt.2025.1622099 (PMC12433952; doi:10.3389/fpsyt.2025.1622099)
Supplement: Supplementary file 1 [file Supplementaryfile1.docx]

Supplementary Material

**Supplementary table 1.** Bibliographic database searches.

| **Databases: Medline, Web of Science, PsycINFO, and Scopus** | |
| --- | --- |
| 1 | Adolescen* [Title/Abstract/Keywords] OR teen* [Title/Abstract/Keywords] OR youth [Title/Abstract/Keywords] OR juvenile [Title/Abstract/Keywords] OR young [Title/Abstract/Keywords] OR minor [Title/Abstract/Keywords] |
| 2 | Game [Title/Abstract/Keywords] OR gaming [Title/Abstract/Keywords] OR gamified [Title/Abstract/Keywords] OR gamification [Title/Abstract/Keywords] OR scape room [Title/Abstract/Keywords] |
| 3 | Intervention [Title/Abstract/Keywords] OR program* [Title/Abstract/Keywords] OR treatment [Title/Abstract/Keywords] OR therapy [Title/Abstract/Keywords] OR trial [Title/Abstract/Keywords] |
| 4 | Class* [Title/Abstract/Keywords] OR school [Title/Abstract/Keywords] OR education institution [Title/Abstract/Keywords] |
| (1 AND 2 AND 3 AND 4) | |

*The following filters were applied in all databases if possible: document types (articles) and languages (English and Spanish).*

| **Medline (*n* = 86)** |
| --- |
| (TI=(adolesc* OR teen* OR "youth" OR "juvenile" OR "young" OR "minor") OR AB=(adolesc* OR teen* OR "youth" OR "juvenile" OR "young" OR "minor") OR AK=(adolesc* OR teen* OR "youth" OR "juvenile" OR "young" OR "minor")) AND (TI=(game OR "gaming" OR "gamified" OR "gamification" OR escape room) OR AB=(game OR "gaming" OR "gamified" OR "gamification" OR escape room) OR AK=(game OR "gaming" OR "gamified" OR "gamification" OR escape room)) AND (TI=(intervention OR program* OR treatment OR therap* OR trial) OR AB=(intervention OR program* OR treatment OR therap* OR trial) OR AK=(intervention OR program* OR treatment OR therap* OR trial)) AND (TI=(class* OR "school" OR "education institution") OR AB=(class* OR "school" OR "education institution") OR AK=(class* OR "school" OR "education institution")) |
| **Web of Science (*n* = 1029)** |
| (TI=(adolescen* OR teen* OR “youth” OR “juvenile” OR “young” OR “minor”) OR AB=(adolescen* OR teen* OR “youth” OR “juvenile” OR “young” OR “minor”) OR AK=(adolescen* OR teen* OR “youth” OR “juvenile” OR “young” OR “minor”)) AND (TI=(game OR “gaming” OR “gamified” OR “gamification” OR escape room) OR AB=(game OR “gaming” OR “gamified” OR “gamification” OR escape room) OR AK=(game OR “gaming” OR “gamified” OR “gamification” OR escape room)) AND (TI=(intervention OR program* OR treatment OR therap* OR trial) OR AB=( intervention OR program* OR treatment OR therap* OR trial) OR AK=(intervention OR program* OR treatment OR therap* OR trial)) AND (TI=(class* OR “school” OR “education institution”) OR AB=(class* OR “school” OR “education institution”) OR AK=(class* OR “school” OR “education institution”)) |
| **PsycINFO (*n* = 15)** |
| (TI adolescen* OR TI teen* OR TI “youth” OR TI “juvenile” OR TI “young” OR TI “minor” OR AB adolescen* OR AB teen* OR AB “youth” OR AB “juvenile” OR AB “young” OR AB “minor”) AND (TI game OR TI "gaming" OR TI "gamified" OR TI "gamification" OR TI escape room OR AB game OR AB "gaming" OR AB "gamified" OR AB "gamification" OR AB escape room) AND (TI intervention OR TI program* OR TI treatment OR TI therap* OR TI trial OR AB intervention OR AB program* OR AB treatment OR AB therap* OR AB trial) AND (TI class* OR TI “school” OR TI “education institution” OR AB class* OR AB “school” OR AB “education institution”) |
| **Scopus (*n* = 22)** |
| TITLE-ABS-KEY((adolesc* OR teen* OR "youth" OR "juvenile" OR "young" OR "minor") AND (game OR "gaming" OR "gamified" OR "gamification" OR escape room) AND (intervention OR program* OR treatment OR therap* OR trial) AND (class* OR "school" OR "education institution")) |

**Supplementary table 2.** QA for single-arm and controlled studies based on the NHLBI assessment tool.

|  | *1* | *2* | *3* | *4* | *5* | *6* | *7* | *8* | *9* | *10* | *11* | *12* | *13* | *14* | Score | *QA* |
| --- | --- | --- | --- | --- | --- | --- | --- | --- | --- | --- | --- | --- | --- | --- | --- | --- |
| Bezençon et al. (2023) | N | Y | CD | N | N | Y | Y | Y | NR | Y | Y | N | Y | Y | 8 | Fair |
| Dietrich et al. (2014) | Y | Y | NR | N | N | Y | Y | NR | NR | Y | N | NA | - | - | 5 | Fair |
| Dietrich et al. (2019) | Y | NR | NR | N | N | Y | Y | NR | NR | Y | N | NA | - | - | 4 | Low |
| Durl et al. (2020) | Y | NR | NR | N | N | NR | NR | NR | NR | Y | Y | N | NR | NR | 3 | Low |
| Galli et al. (2023) | N | N | N | N | N | Y | Y | Y | NR | Y | Y | N | NA | Y | 6 | Fair |
| Guldager et al. (2022) | Y | Y | CD | N | N | Y | N | Y | NR | Y | Y | Y | Y | Y | 9 | Fair |
| McMahon & Hanrahan (2018) | N | N | N | NR | N | Y | Y | Y | Y | CD | Y | N | Y | Y | 7 | Fair |
| Perry et al. (2009) | Y | NR | CD | N | N | Y | Y | Y | Y | Y | Y | N | Y | Y | 9 | Fair |
| Rundle-Thiele et al. (2013) | Y | Y | NR | NR | NR | Y | Y | NR | Y | Y | N | NA | - | - | 6 | Fair |
| Rundle-Thiele et al. (2015) | Y | Y | Y | N | N | Y | N | N | NR | Y | Y | N | NR | Y | 7 | Fair |
| Sidhu et al. (2016) | N | N | N | N | N | Y | N | N | NR | Y | Y | N | Y | Y | 5 | Low |
| Stein-Seroussi et al. (2009) | Y | Y | NR | N | N | Y | Y | Y | NR | Y | N | N | Y | Y | 8 | Fair |
| Stigler et al. (2007) | Y | NR | NR | N | N | Y | N | Y | Y | Y | Y | N | Y | Y | 8 | Fair |
| Sussman et al. (2001) | Y | Y | CD | N | N | Y | N | Y | N | Y | Y | N | NA | Y | 7 | Fair |
| Tuijnman et al. (2022) | Y | NR | CD | N | N | N | Y | Y | Y | Y | N | Y | NA | Y | 7 | Fair |
| Wodarski (1987) | N | NR | CD | N | N | Y | Y | Y | NR | Y | Y | N | NA | Y | 6 | Fair |
| Zarshenas et al. (2020) | Y | NR | CD | N | N | Y | NR | NR | NR | Y | Y | N | NA | Y | 5 | Low |

***Note***: Y, Yes; N, No; CD, cannot determine; NA, not applicable; NR, not reported. Scores: 1 = Yes; 0 = no, cannot be determined, not applicable, or not reported. The total score range was 0-14 for the “Quality Assessment of Controlled Intervention Studies” tool: good QA (11-14), fair QA (6-10), and low QA (≤ 5); and 0-12 for the “Quality Assessment Tool for Before-After Studies with No Control Group” tool: good QA (9-12), fair QA (5-8), and low QA (≤ 4). The tool and its items can be found here: <https://www.nhlbi.nih.gov/health-topics/study-quality-assessment-tools>
